# Health-Related Effects Reported by Electronic Cigarette Users in Online Forums

**DOI:** 10.2196/jmir.2324

**Published:** 2013-04-08

**Authors:** My Hua, Mina Alfi, Prue Talbot

**Affiliations:** ^1^University of CaliforniaDepartment of Cell Biology and NeuroscienceUniversity of CaliforniaRiverside, CAUnited States

**Keywords:** Electronic cigarettes, e-cigarettes, electronic nicotine delivery devices, ENDS, health effects, nicotine, harm reduction, symptoms, Internet

## Abstract

**Background:**

The health effects caused by electronic cigarette (e-cigarette) use are not well understood.

**Objective:**

Our purpose was to document the positive and negative short-term health effects produced by e-cigarette use through an analysis of original posts from three online e-cigarettes forums.

**Methods:**

Data were collected into Microsoft Access databases and analyzed using Cytoscape association graphics, frequency distributions, and interactomes to determine the number and type of health effects reported, the organ systems affected the frequency of specific effects, and systems interactions.

**Results:**

A total of 405 different symptoms due to e-cigarette use were reported from three forums. Of these, 78 were positive, 326 were negative, and one was neutral. While the reported health effects were similar in all three forums, the forum with the most posts was analyzed in detail. Effects, which were reported for 12 organ systems/anatomical regions, occurred most often in the mouth and throat and in the respiratory, neurological, sensory, and digestive systems. Users with negative symptoms often reported more than one symptom, and in these cases interactions were often seen between systems, such as the circulatory and neurological systems. Positive effects usually occurred singly and most frequently affected the respiratory system.

**Conclusions:**

This is the first compilation and analysis of the health effects reported by e-cigarette users in online forums. These data show that e-cigarette use can have wide ranging positive and negative effects and that online forums provide a useful resource for examining how e-cigarette use affects health.

## Introduction

New nicotine delivery products, often advertised as “safer” or with “harm reduction”, are designed to reduce health problems caused by cigarette smoking [1,2]. Electronic cigarettes, also called e-cigarettes or electronic nicotine delivery systems, are one such product that has rapidly gained popularity worldwide [3,4]. E-cigarettes produce aerosol by heating a humectant (usually propylene glycol or vegetable glycerol) containing nicotine and flavorings without actually burning tobacco. The aerosol, when inhaled, delivers nicotine to the user [5,6]. Users can purchase either prepackaged cartridges/cartomizers with varying amounts of nicotine or bottles of fluid for refilling empty cartridges/cartomizers [7]. E-cigarettes vary widely in their performance with respect to pressure drop, air flow rate required to activate the battery, aerosol delivery, number of puffs produced per cartridge, and in the amount of nicotine/puff [7-9]. Puff duration data mined from YouTube videos showed that e-cigarette users on average take puffs that are over twice as long as conventional smokers [10]. Because e-cigarette production is currently not regulated, quality control during manufacture has been questioned [11-14].

While e-cigarettes may facilitate smoking cessation [15-17], users are concerned about product safety and toxicity [5]. Most knowledge regarding the health effects of e-cigarette use comes from studies on the emissions in their aerosol [14,18]. Because e-cigarettes deliver fewer total chemicals and fewer carcinogens than conventional tobacco-burning cigarettes, they are sometimes considered safer products [18,19]. However, e-cigarette cartridge fluids and their emissions are not yet well characterized and may vary among products. In a recent study, e-cigarette refill fluids varied significantly in their cytotoxicity when tested *in vitro* with embryonic and adult cells [20]. One cartridge analyzed by the US Food and Drug Administration contained diethylene glycol, a known toxicant [21], and errors in labeling of nicotine concentrations have been reported [12,14]. Moreover, performance of e-cigarettes is highly variable both between and within brands. A recent study of e-cigarette users showed that 5 minutes of puffing adversely affected lung physiology [22,23], indicating that a better understanding of the health effects related to e-cigarette use is needed.

The Internet has become a useful resource for consumers seeking health information online. Moreover, Internet sites can be mined to acquire data relevant to issues dealing with public health, a science referred to as infodemiology [24-26]. Several prior infodemiological-type studies have produced informative data related specifically to tobacco products [3,10,27]. E-cigarette forums have an increasingly popular presence on the Internet where e-cigarette users can post data relevant to the health effects they experience from using these products. In this study, we used an infodemiological approach to acquire and analyze self-reported health effects posted on Internet forums by e-cigarette users. While medical and lifestyle histories are not known for the individuals in this study, these Internet data indicate that a broad spectrum of symptoms may accompany e-cigarette use and that further studies are needed in this area.

## Methods

A Google Internet search was performed using the words “electronic cigarette forum” to identify online e-cigarette forums with “health and safety sections” that allowed posts on the health effects experienced when using e-cigarettes. The three websites with the highest number of posts in health and safety sections were selected for study (Electronic Cigarette Forum posts = 543, Vapers Forum posts = 34, and Vapor Talk posts = 55). Data were collected from posts on these websites through July 15, 2011, and the Electronic Cigarette Forum, which had the most entries, was analyzed in detail. Only data reported directly by an e-cigarette user were included.

Databases were created using Microsoft Access to record basic information (age, gender, location) and positive and negative health effects. These data were recorded for persons who reported their own health effects and excluded those that reported health effects of others. Basic user information was gathered by accessing the individual’s profile pages upon entering their posts. Data were also recorded for users who visited a physician/dentist or emergency room and self-reported their diagnosis on a forum website. These entries were recorded as “signs”.

To ensure health reports were not duplicated or over-reported, individual symptoms that users reported were grouped under their coded user name. Any duplicate reports of symptoms were omitted and counted only once.

Data were analyzed iteratively. In the first analysis, all health-related effects reported by e-cigarette users were grouped according to the organ system/anatomical region, which we define as systems. Some effects, such as improved overall health, could not be categorized by system and were kept separately. When users described their effects with synonyms (eg, fatigue and lethargy), the effects were combined using one term, such as fatigue. Sometimes symptoms were characterized by degree, such as severe stomach cramps or stomach cramps, in which case the symptoms were grouped in the category “stomach cramps”. When a symptom could have been associated with more than one system, the effect was assigned to the system for which it had the strongest fit (eg, improved sense of taste was assigned to sensory but could have been mouth/throat). An association graph was created using Cytoscape software (an interactome creation and analysis program) to group the health effects by systems.

Following the initial analysis, health-related effects were consolidated within a system to make the data more manageable. For example, there were many symptoms involving the tongue, such as swollen tongue, red tongue, and bumps on tongue. These were all grouped under “tongue” for further analysis. Frequency distributions for the grouped data in each system were plotted using MS Excel.

Information pertaining to individual users and the systems affected by e-cigarette use was transferred from Access to Excel spreadsheets and uploaded to Cytoscape software. Interactomes were then created using the edge-weighted spring embedded or force directed views.

## Results

### Demographics

The Electronic Cigarette Forum had the largest number of users (Table 1). Because some of these users posted multiple times, the total number of health-related posts attributed to e-cigarettes (N=543) was greater than the total number of users (N=481). Most users posted symptoms only (n=492) (ie, health effects perceived by the individual), while some reported both symptoms and signs (n=20) (ie, effects diagnosed by a physician then self-reported in the forum by the user), or signs only (n=31). Users’ ages ranged from 18-71 years with the highest number being in the 26-35 year age bracket. For the few users that provided gender on the Electronic Cigarette Forum, more females self-reported health effects than males. Most users were from North America. Demographics for the two smaller forums (Vapers Forum and Vapor Talk) are also provided in Table 1.

**Table 1:** Demographics of forum users.

Characteristics			ECF^a^	VF^b^	VT^c^
**Self-reported ages in years**					
	18-25		46	N/A	N/A
	26-35		107	N/A	8
	36-45		60	1	8
	46-55		31	1	4
	>56		17	N/A	1
	Did not state		220	29	27
**Gender**					
	Male		10	N/A	24
	Female		24	N/A	14
	Did not state		447	31	10
**Location**					
	North America				
		US Midwest	60	4	8
		US Northeast	70	2	7
		US Southeast	84	5	10
		US Southwest	30	N/A	4
		US West	69	5	11
		US territories	2	N/A	N/A
		US unspecified	18	N/A	N/A
		Canada	14	2	3
	Europe				
		UK	13	N/A	N/A
		Ireland	3	N/A	N/A
		Other	6	N/A	N/A
	Other regions				
		Africa	4	N/A	N/A
		Australia, New Zealand	7	1	N/A
		Asia	6	N/A	N/A
		Central America	1	N/A	N/A
	Did not state		94	12	5
**Total number users evaluated**			481	31	48
**Total number of posts**			543	34	55

^a^ ECF = Electronic Cigarette Forum

^b^ VF = Vapers Forum

^c^ VT = Vapor Talk

### Health Effect-System Associations

**Figure 1:**
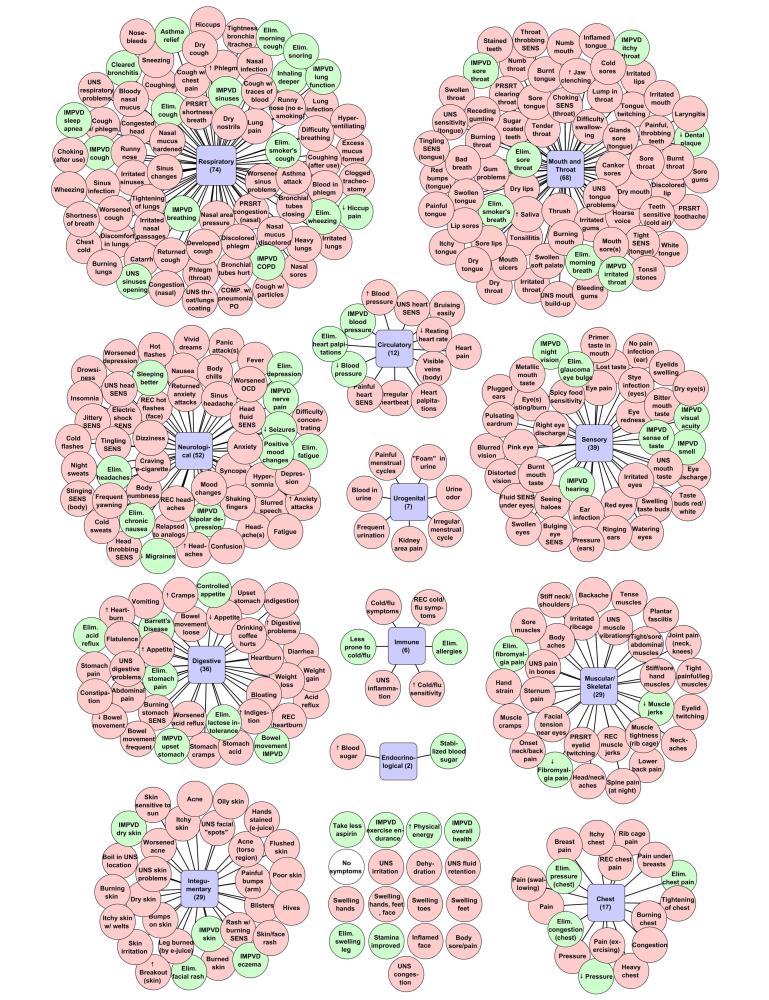
Summary of all positive (green), negative (red),and neutral (white) symptoms and their associated systems as reported by e-cigarette users on the Electronic Cigarette Forum.

A total of 388 different symptoms were reported by e-cigarette users on the Electronic Cigarette Forum (Figure 1). Most health effects were broadly distributed across 12 different categories. These categories included 10 organ systems (eg, respiratory, neurological) and two anatomical regions (chest and mouth/throat), which we collectively refer to as systems. Respiratory, mouth/throat, neurological, and sensory had the most symptoms associated with them. Mouth and throat had more negative symptoms than any other group. A significant number of health effects appeared in the digestive, muscular/skeletal, and integumentary systems, while the urogenital, immune, and endocrinological systems had relatively few symptoms. Seventeen reported symptoms were not associated with a system. (The legend for Figure 1 is as follows: IMPVD = improved; Elim = eliminated; REC = recurring; PRSRT = persistent; UNS = unspecified; SENS = sensation; OCD = obsessive compulsive disorder; COPD = chronic obstructive pulmonary disease; COMP = complications; PO = post-operative; w/ = with.)

Of the reported effects, 318 were negative, 69 were positive, and one was neutral (Figure 1). All systems had both positive and negative symptoms, except for urogenital, which had only negative effects. Negative symptoms were often described as persistent, worsened, or increasing. In contrast, positive effects were often described as decreased, improved, or eliminated. Negative and positive effects were sometimes opposites, eg, improved cough and worsened cough were reported by different individuals. Some symptoms (17) were not assigned to a category. Negative symptoms in this group included “swelling” and “dehydration”, while positive included “improved stamina” and “improved overall health”. Some symptoms occurred during e-cigarette use, such as “metal taste in mouth”, while others occurred after use, such as “choking after use”. Of the symptoms not associated with a system, the most frequently reported were: no symptoms [4], improved exercise endurance (6), and dehydration (7).

### Symptom Frequency

**Figure 2:**
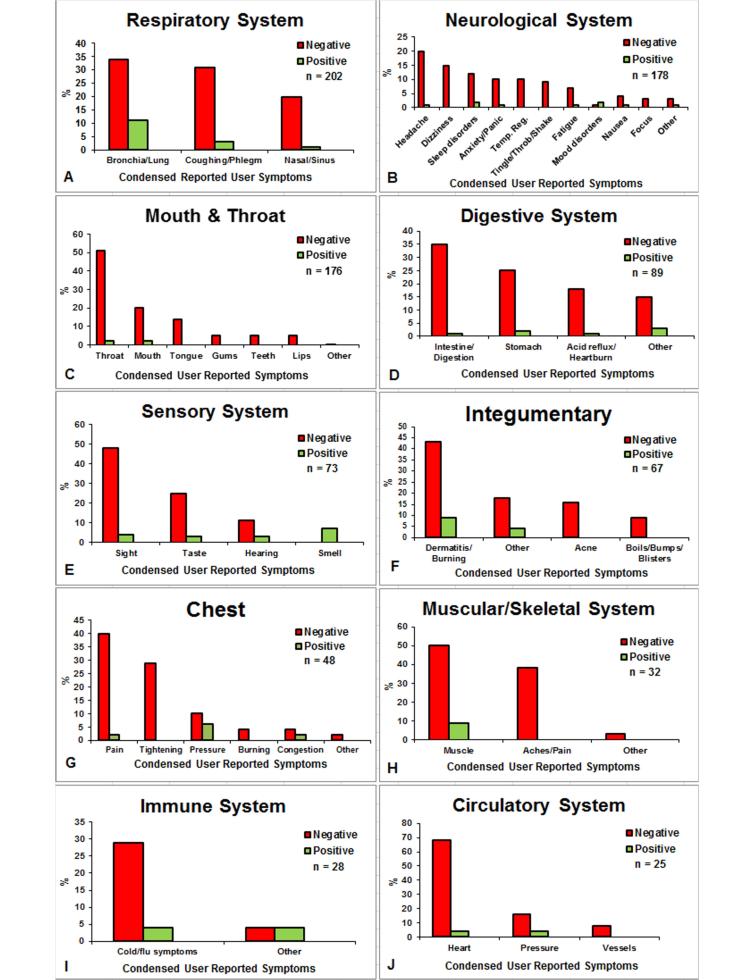
Frequency of positive and negative symptoms for 10 systems.

To analyze the frequency of reports for various symptoms, the data in Figure 1 were condensed by combining all health effects into structural or physiological groups. For example, red tongue, swollen tongue, and bumps on tongue were all combined into the structural group “tongue”, and constipation, indigestion, and frequent bowel movements were combined into the physiological group “intestine/digestion”. The frequency of positive and negative reports in each structural/physiological group was graphed for each system (Figure 2). In Figure 2, n=the total number of health effects (positive and negative) for each system, and data in each column are plotted as a percentage of the total number of effects reported for each system. Urogenital and endocrinological data are not shown because there were few reports in these categories. For each system in Figure 2, the negative effects outnumbered the positive effects. In most graphs, there were one or two negative structural/physiological groups that were dominant. For example, the bronchi/lungs were frequently affected in “respiratory”, and sight was frequently affected in “sensory”. Some structural/physiological groups had only negative effects, such as acne in “integumentary”, tongue in “mouth and throat”, and aches/pain in “muscular/skeletal”. In respiratory, digestive, sensory, and immune, each condensed group had both positive and negative symptoms. One physiological group, smell in “sensory”, had only positive reports. The neurological system had the largest number of different groups, which included headaches, dizziness, and temperature regulation. 

### Interactomes

**Figure 3:**
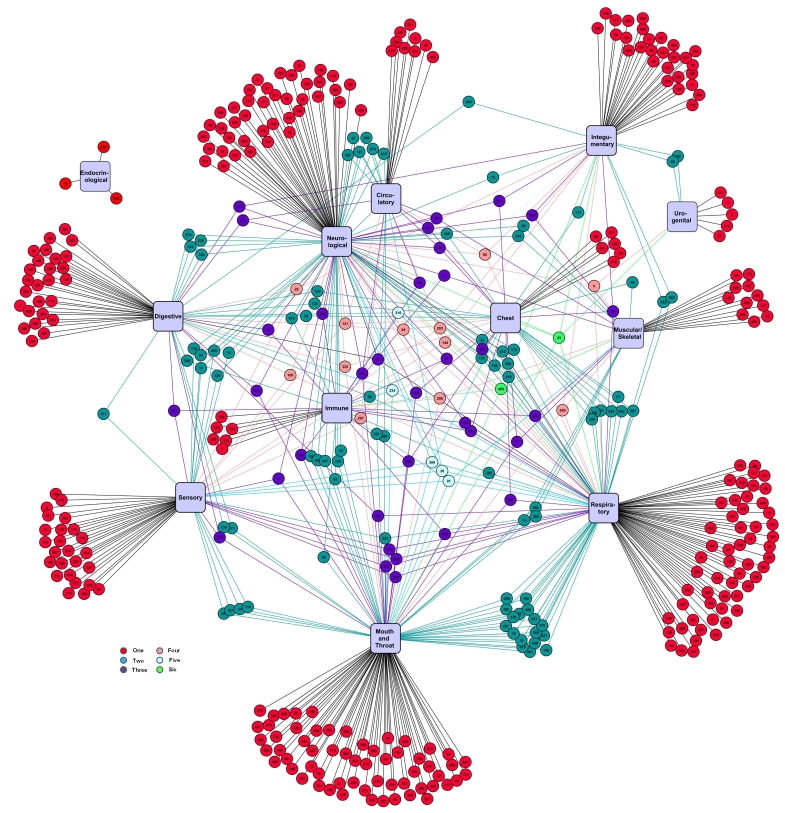
Interactome showing relationship between users who reported negative symptoms and the systems that were affected.

Electronic Cigarette Forum data were further analyzed by creating an interactome showing the relationship between the negative symptoms reported by each individual and the systems with which they interacted (Figure 3). Individuals in the interactome are represented by small numbered circles, while systems appear as rectangular purple nodes. Those reporting symptom(s) for one system (n=276, red circles) are generally at the outer edges of the interactome. Individuals reporting two systems (n=85, teal circles) are grouped more toward the center of the interactome or between adjacent systems with which they interact. Those reporting health effects in three (n=34, purple circles), four (n=12, pink circles), five (n=5, light blue circles), or six (n=2, green circles) systems are grouped in the interior of the interactome.

For most users, a single system was affected. However, 34% of the individuals had negative effects in more than one system. For individuals reporting effects in two systems, clear interactions were seen between: mouth/throat and respiratory; respiratory and chest; mouth/throat and sensory; sensory and neurological; mouth/throat and neurological; neurological and respiratory; digestive and neurological, and neurological and circulatory. Although a number of individuals reported symptoms in immune, there was no clear interaction between immune and other systems. There was relatively little interaction with urogenital and no interaction with endocrinological.

**Figure 4:**
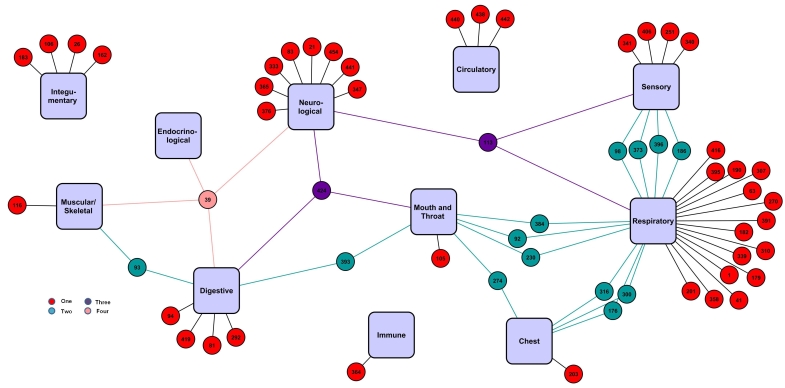
Positive interactome for the Electronic Cigarette Forum.

The corresponding positive interactome for the Electronic Cigarette Forum had relatively few users (n=58) (Figure 4). Two-way interactions were seen mainly between respiratory and sensory, respiratory and mouth/throat, and respiratory and chest. For most users, a single system was affected, although 2 users reported positive effects in as many as four different systems.

The color-coding in Figure 4 shows the number of systems affected for each user (red = users reporting symptom(s) for one system; teal = users reporting symptom(s) for two systems; purple = users reporting symptom(s) for three systems; pink = users reporting symptom(s) for four systems).

### Time Frame

In the Electronic Cigarette Forum, 66 users reported a time frame for the appearance of their symptoms. Forty-seven users stated that symptoms occurred 1 week or less after use began. The remaining 19 reported that symptoms occurred more than 1 week after use began.

### Signs

Some e-cigarette users reported diagnoses (signs) made by physicians or dentists (Multimedia Appendix 1). Most signs occurred in the circulatory system, respiratory system, or mouth/throat. Several negative health effects that did not appear in the self-reported data were diagnosed by physicians and dentists, such as periodontitis, rhinitis, parethesia, cataract development, and anemia. Some positive health effects that were diagnosed but did not appear in user-reported symptoms (Figure 1) were whiter teeth, improved gum health, and improved spirometry test results.

### Other Forums

**Figure 5:**
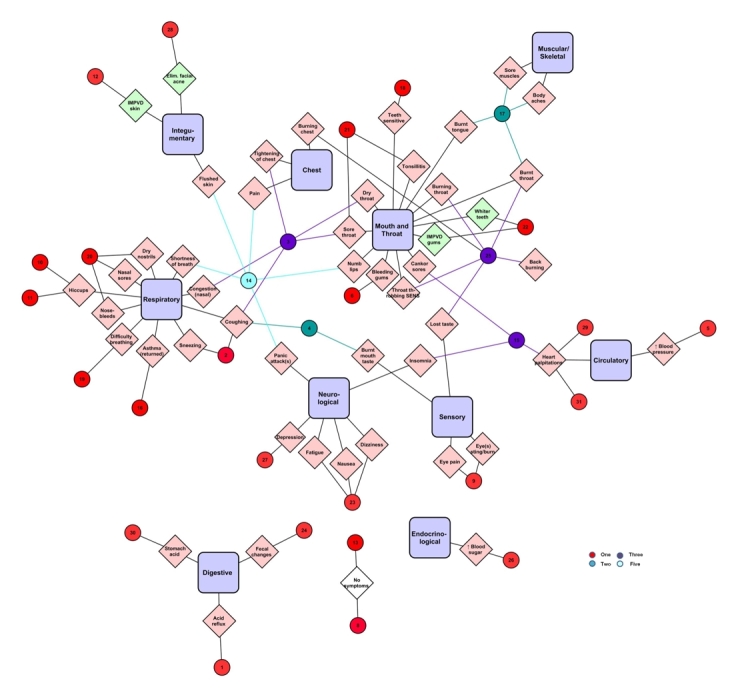
Interactome for Vapers Forum.

**Figure 6:**
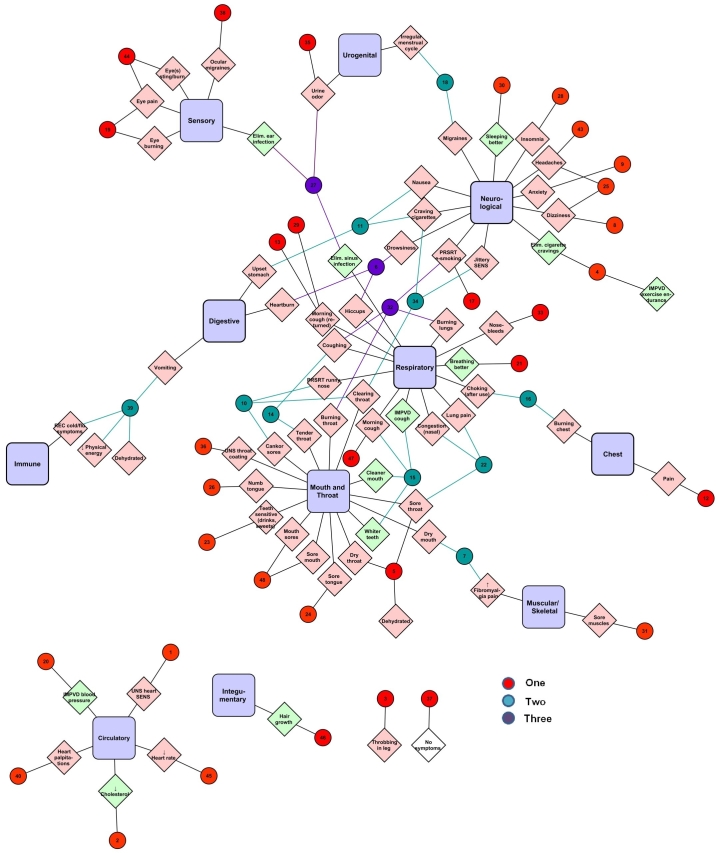
Interactome for Vapor Talk.

Interactomes for the Vapor Talk (Figure 5) and Vapers Forums (Figure 6) show the relationship between users (small circles), negative symptoms (light pink diamonds) and positive symptoms (light green diamonds), and systems (purple rectangles). Data for these smaller forums were similar to the Electronic Cigarette Forum. In both small forums, more negative than positive symptoms were posted, and most effects were reported for “respiratory”, “mouth/throat”, and “neurological”. Most users reported only one effect that generally appeared near the outside edge of the interactome. Those reporting two and or more effects are grouped toward the center of the interactome. There were not enough individuals in the two smaller forums to observe interactions. Very few individuals in the two smaller forums reported signs (Multimedia Appendix 1). The color-coding in Figures 5 and 6 shows the number of systems affected for each user (red circles = users reporting symptom(s) for one system; teal circles = users reporting symptom(s) for two systems; purple circles = users reporting symptom(s) for three systems; light blue = users reporting symptom(s) for five systems).

## Discussion

Information on the health effects related to e-cigarette use is important to users, health professionals, and regulatory agencies. In this study, we have taken an infodemiological approach (ie, used information on the Internet for public health research) [26] to evaluate the health effects reported by e-cigarette users. Our data, while preliminary, show that a broad spectrum of positive and negative symptoms attributed to e-cigarette use has been self-reported in online forums. Our data are in general agreement with prior studies and extend earlier work by demonstrating the breadth of e-cigarette related health effects, determining the frequency of occurrence of specific effects for 12 systems, and demonstrating interactions between systems.

Two prior studies deal with the effects of e-cigarette use on blood pressure and heart rate. When 32 participants consumed one e-cigarette cartridge/day for 4 weeks, no abnormal changes in blood pressure were observed [28]. In contrast, we found, based on a larger number of participants, that blood pressure changes were reported by 3.5% of e-cigarette users (5 individuals with symptoms and 12 individuals with signs), and increased blood pressure was the sign most frequently diagnosed in e-cigarette users by physicians (n=9). Increased heart rate was observed in a study in which increased levels of plasma nicotine were verified during e-cigarette use [6]. This agrees with our study in which some e-cigarette users reported changes in heart rate and palpitations. Although we found that e-cigarette users reported relatively few different symptoms for the circulatory system, some, such as increased blood pressure, may not be perceived by the user and could have significant health impacts if left untreated.

Several prior health-related studies on e-cigarettes deal with the respiratory system and mouth/throat. In our study, these systems had the largest number of different symptoms associated with them. In an Internet survey of e-cigarette users, positive effects were reported to be improved breathing/respiration, less coughing, fewer sore throats, improved fitness, and reduced bad breath [5], similar to the main positive effects found in our study. We found additional positive effects such as “improved COPD [chronic obstructive pulmonary disease]”, “asthma relief”, and “cleared bronchitis”. However for most positive effects, there were corresponding negative reports, such as “developed cough” or “cough worsened”, suggesting that individual responses to e-cigarette aerosol can vary and be opposite. In a study of smoking reduction, mouth and throat irritation as well as dry cough were common, but diminished by 24 weeks [29]. These symptoms were reported in our study with high frequency. We found that some users had immediate reaction to e-cigarettes, such as coughing or choking, or same-day reaction (insomnia), while others reported that symptoms appeared at least a week after beginning e-cigarette use. When respiratory physiology was monitored following 5 minutes of ad libitum use of e-cigarettes by healthy non-smokers, a significant decrease in exhaled nitric oxide and an increase in pulmonary resistance were observed, similar to effects seen during use of conventional cigarettes [22,23]. We did find reports of “tightening of the lungs” and “difficulty breathing”, which suggest increased pulmonary resistance. In all the above studies, the reported health effects are short term.

In general, the negative short-term effects reported by e-cigarette users appear relatively minor compared to more serious long-term conditions (eg, cancer and stroke) that occur in conventional smokers [30]. Since some symptoms associated with e-cigarette use appear to be minor, such as sneezing and dry skin, and may not impact overall health and life style, e-cigarette users may consider these effects to be insignificant compared to the health consequences of conventional smoking. Other effects correlated with e-cigarette use may forewarn of significant health problems. For example, dizziness could have a major impact on a user’s life style if it were serious enough to prevent driving or working, as was reported to us by an e-cigarette user. Users need to be aware that e-cigarettes are not free of health consequences and that numerous negative effects have been reported. For individuals who do not smoke conventional cigarettes and who do not currently use e-cigarettes, knowledge of these health effects may be important in helping them decide if they wish to begin e-cigarette use.

Many e-cigarette users reported multiple positive or negative symptoms that often affected more than one system, as shown in Figures 3 and 4. The negative interactome for the Electronic Cigarette Forum demonstrated that when multiple systems are affected in individual e-cigarette users, there is often interaction between systems, such as respiratory and chest, respiratory and mouth/throat, and digestive and neurological. This information may be useful to physicians treating patients who are using e-cigarettes and/or have a prior history of smoking. The data may also be used to help diagnose health problems in patients for whom smoking/e-cigarette history is not known. Although the positive interactome for the Electronic Cigarette Forum had fewer users, interactions between systems were similar to those observed in the negative interactome.

In the negative Electronic Cigarette Forum interactome, many symptoms involved the neurological and sensory systems, and these may have been caused by an overdose or withdrawal of nicotine, which activates nicotinic cholinergic receptors in the brain [31]. Some symptoms reported by e-cigarette users, such as vomiting, nausea, changes in heart rhythm, confusion, dizziness, and fatigue, would be consistent with a nicotine overdose [32]. Because nicotine can be added to cartomizers/cartridges, the potential to overdose exists. It is also possible that some reported symptoms, such as anxiety and depression, which are characteristic of nicotine withdrawal [33], were caused by insufficient delivery of nicotine to some e-cigarette users. As previously shown, inexperienced users of e-cigarettes may not receive adequate levels of nicotine [34].

While our study demonstrates the value of an infodemiological approach, it has several limitations. The online forums may be biased toward negative reporting, and the positive effects are probably under-reported in our analysis. In the current study, users posted effects that they observed after starting e-cigarette use and that they therefore attribute the effects to e-cigarettes. However, these results do not take into account past smoking and lifestyle history, pre-existing conditions, or other medical problems the users may have had before e-cigarette use began, any of which may have affected or even aggravated their response to e-cigarette aerosol. In addition, user posts cannot be validated, and it is possible that inaccurate information appears in the forums. Future work should be undertaken using methods that would acquire additional medical and lifestyle history of each user and that draw participants from a random sample of e-cigarette users.

Additionally, our data do not address the effects that e-cigarette use may have on prenatal development, a period in the human life cycle that is particularly sensitive to environmental chemicals [35,36]. Our recent study found that some e-cigarette refill fluids were highly toxic to human embryonic stem cells [20], indicating the need for further work in this area.

Our data will be helpful to e-cigarette users who may experience similar effects, to health care professionals advising individuals on e-cigarette usage, and to policy makers and legislators who regulate sales, use, and marketing of e-cigarettes. Interactome data may help health care workers identify problems caused by e-cigarette use, eg, problems with the respiratory system are often linked to problems with the neurological system. While it can be argued that e-cigarettes are safer than conventional tobacco-burning products, the data in this study demonstrate that e-cigarette users are not free of negative health effects.

### Conclusions

This study provides a preliminary synopsis of the short-term health effects related to e-cigarette use as reported in online forums. Effects occurred most often in the respiratory and neurological systems and in the mouth and throat with a total of 12 systems being affected. Within each system, certain categories of health related-effects were dominant, such as throat problems and headache. As more individuals adopt e-cigarettes and use them for longer periods of time, additional positive and negative effects on human health will likely be reported. This study is a step in understanding these issues; however, it will be many years before the long-term health consequences of e-cigarette use are known.

## Multimedia Appendix 1

Supplementary table.
