# Supplementary material for: Health-Related Effects Reported by Electronic Cigarette Users in Online Forums
Source: J Med Internet Res. 2013 Apr 8;15(4):e59. doi: 10.2196/jmir.2324 (PMC3636314; doi:10.2196/jmir.2324)
Supplement: Supplementary file 1 [file jmir_v15i4e59_app1.pdf]

Multimedia Appendix 1

Supplementary Table 1: Reported Signs

	Frequency of Reports		
	ECF[1]	VF[2]	VT[3]
**Systems**			
**Circulatory**			
Increased Blood Pressure	9	/	/
Normal Blood Pressure	5	1	/
Decreased Blood Pressure	3	/	/
Increased pulse/heart rate	3	/	/
Heart Murmur	1	/	/
Increased cholesterol	1	/	/
High Nicotine level in blood test	/	1	/
**Respiratory**			
Wheezing	3	/	/
Asthma attack	2	/	/
Improved spirometry test/reading	2	/	/
Mycoplasma pneumonia	1	/	/
Eliminated wheezing	1	/	/
Rhinitis	1	/	/
Sinus infection	1	/	/
Streptococcal nose infection			
**Mouth and Throat**			
Abscessed tooth	2	/	/
Bleeding gums	2	/	/
Chapped lips	/	/	/
Improved gum health	2	/	/
Infected root canal	1	/	/
Less plaque	1	/	/
Periodontitis	1	/	/
Tooth cavity	1	/	/
Whiter teeth	1	/	/
Thrush in throat and sinuses	1	/	/
Sore throat	/	/	1
Stone in salivary gland	/	/	1
**Neurological**			
Parethesia	1	/	/
Anxiety/Panic attack	2	/	/
**Sensory**			
Eye resting dilation worse	1	/	/
Cataract development	1	/	/
**Immune**			
Anemia	1	/	/
Allergies	1	/	/
Persistent flu	/	/	1
**Muscular-Skeletal**			
Spinal Osteoarthritis	1	/	/
**Integumentary**			
Eczema	1	/	/

[1] ECF = Electronic Cigarette Forum
[2.] VF = Vapers Forum
[3.] VT = Vapor Talk
